# Imperatorin Improves Obesity-Induced Cardiac Sympathetic Nerve Injury Mediated by P2X4 Receptor in Stellate Sympathetic Ganglion

**DOI:** 10.3390/ijms24010783

**Published:** 2023-01-02

**Authors:** Mingming Zhang, Yuqing Wen, Peiwen Liang, Changsen Yang, Hongcheng Tu, Jingyi Wei, Junpei Du, Ting Zhan, Shangdong Liang, Guodong Li, Yun Gao

**Affiliations:** 1 Department of Physiology, Basic Medical College, Nanchang University, Nanchang 330006, China; 2 Second Clinic Medical College, Nanchang University, Nanchang 330006, China; 3 Joint Program of Nanchang University and Queen Mary University of London, Nanchang University, Nanchang 330006, China; 4 Basic Medical College, Nanchang University, Nanchang 330006, China; 5 Jiangxi Provincial Key Laboratory of Autonomic Nervous Function and Disease, Nanchang 330006, China

**Keywords:** obesity, P2X4 receptor, sympathetic excitement, stellate sympathetic ganglia, pyroptosis, imperatorin

## Abstract

Obesity can activate the inflammatory signal pathway, induce in the body a state of chronic inflammation, and increase the excitability of the sympathetic nervous system, which may induce sympathetic neuropathic injury. The stellate sympathetic ganglia (SG) can express the P2X4 receptor, and the abnormal expression of the P2X4 receptor is related to inflammation. Imperatorin (IMP) is a kind of furan coumarin plant which has anti-inflammatory effects. This project aimed to investigate whether IMP can affect the expression of P2X4 receptors in the SG of obese rats to display a protective effect from high-fat-triggered cardiac sympathetic neuropathic injury. Molecular docking through homology modelling revealed that IMP had good affinity for the P2X4 receptor. Our results showed that compared with the normal group, the administration of IMP or P2X4 shRNA decreased sympathetic excitement; reduced the serum levels of triglyceride, total cholesterol, and lactate dehydrogenase; downregulated the expression of P2X4 receptors in SG; and inhibited the expression of inflammatory factors in the SG and serum of obese rats significantly. In addition, the expression of factors associated with the cell pyroptosis GSDMD, caspase-1, NLRP-3, and IL-18 in obese rats were significantly higher than those of the normal rats, and such effects were decreased after treatment with IMP or P2X4 shRNA. Furthermore, IMP significantly reduced the ATP-activated currents in HEK293 cells transfected with P2X4 receptor. Thus, the P2X4 receptor may be a key target for the treatment of obesity-induced cardiac sympathetic excitement. IMP can improve obesity-induced cardiac sympathetic excitement, and its mechanism of action may be related to the inhibition of P2X4 receptor expression and activity in the SG, suppression of cellular pyroptosis in the SG, and reduction of inflammatory factor levels.

## 1. Introduction

Obesity has become a worldwide epidemic, and as the number of obese people increases, hyperlipidaemia, metabolic syndrome, and especially cardiovascular diseases (CVDs) are becoming major health risks [1]. Obesity and its complications, such as insulin resistance (IR), type 2 diabetes, and cardiovascular disease, are associated with a state of chronic inflammation and oxidative stress [2]. The production of compounds by immune cells during obesity leads to a vicious cycle of increased inflammation, oxidation, and harmful effects, resulting in increased tissue damage and immune system dysfunction [3]. 

In recent years, the role of purinoceptors in the central nervous system has attracted the interest of researchers. Purinoceptors are divided into P1 and P2 receptors [4]. P2X receptors are involved in the transmission of injurious messages [5]. The P2X4 receptor is a cation-selective ion channel receptor with a relatively high permeability to Ca^2+^. It has been found that the activation of P2X4 receptors of the microglia in the dorsal horn of the spinal cord can release various inflammatory substances, such as IL-1β, TNF-α, and neuroactive substances [6,7]. Peripheral nerve injury has been reported to produce inflammatory factors through the activation of microglia in the dorsal horn of the spinal cord, causing inflammatory pain through the activation of glial cell P2X4 receptors, suggesting that P2X4 receptors may be an important target for the treatment of chronic inflammatory lesions [6,8]. The satellite glial cells of the stellate sympathetic ganglion (SG) can express P2X4 receptors and receive signals from myocardial damage [9]. The P2X4 receptors in the satellite glial cells of SGs may be involved in the impairment of cardiac sympathetic nerve function in response to obesity because it was reported that increased P2X4 receptors in SGs may contribute to the diabetic cardiac autonomic neuropathy [10].

Obesity increases the release of substances that promote the excitability of the sympathetic nervous system, and abnormal excitation of the sympathetic nervous system can be an important cause of IR [11]. Sympathetic ganglion neurons are surrounded by satellite glial cells, which together form a special unit [12]. During nerve injury, glial cells release a variety of inflammatory factors that act directly on neuron surface receptors for development and maintenance of chronic inflammation [13]. When obesity triggers neurological damage to the body, it has been shown that cell pyroptosis is also involved [14]. The process of cell pyroptosis is an enzymatic cascade reaction of the caspase family. Caspase-1, the first caspase discovered, is a pro-inflammatory cellular protein that mediates the maturation of specific cytokines [15]. Caspase-1 can use GSDMD as a substrate for cleavage of both IL-1β/18 cytokines, promoting their maturation and secretion. It has been demonstrated that GSDMD is an executing protein that initiates the cell pyroptosis program. The activated caspase-1 cleaves the junction between the N-terminal and C-terminal structural domains of GSDMD, releasing the N-terminal structural domain, which fulfils the role of binding membrane phospholipids upon membrane perforation, thus disrupting the cell membrane and triggering cell pyroptosis [16]. In addition, it has also been shown that the NLRP family plays an important role in cell pyroptosis through the NLRP3/caspase-1 inflammasome signaling pathway [17]. A previous study in our lab showed that silencing the P2X4 receptor may act effectively against gp120-induced pyroptosis in dorsal root ganglion [18]. Thus, we speculate that the P2X4 receptor may be implicated in the pyroptosis induced by obesity in the SG.

Imperatorin is a secondary product of a plant belonging to the furanocoumarins group with anti-tumor, anti-inflammatory, analgesic, cardiovascular, and neurological modulating effects [19,20]. In this study, we prepared an obese rat model to observe the effects and underling mechanisms of imperatorin application on P2X4 receptor-mediated pathological changes in the SG. It is hoped that this project will broaden and deepen the understanding of the pathogenesis of sympathetic neuropathy caused by obesity and explore new avenues for its prevention and treatment.

## 2. Results

### 2.1. Molecular Docking

**Figure 1:**
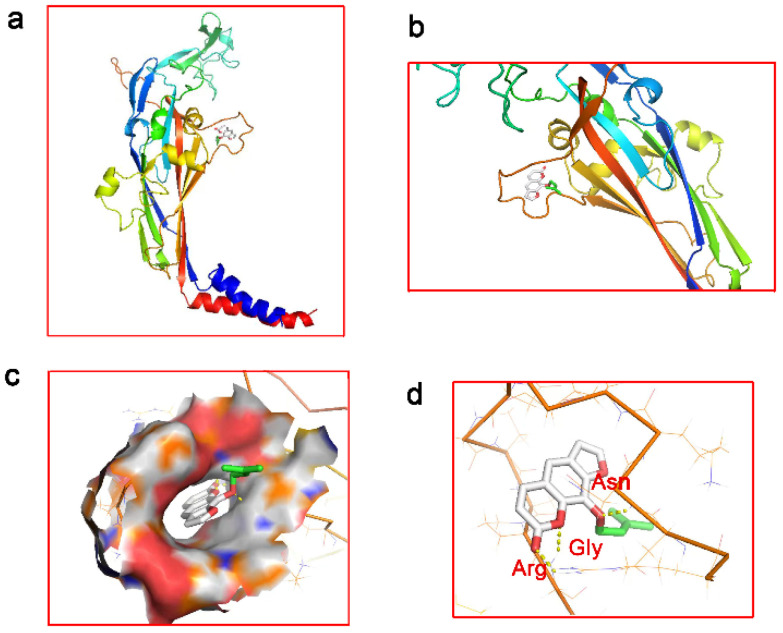
Molecular docking of P2X4 and imperatorin: (**a**) the frontal view of the P2X4 receptor binding to imperatorin; (**b**) the top view of the P2X4 receptor binding to imperatorin; (**c**) the ligand-binding pocket view of the P2X4 receptor binding to imperatorin; and (**d**) the view of P2X4 different molecules binding to imperatorin.

Molecular docking results showed that the P2X4 receptor had a strong binding ability with imperatorin, and ARG, GLY, and ASN amino acid residues were the best binding sites for the P2X4 receptor to imperatorin (Figure 1 and Table 1).

### 2.2. The Behavioral Change

#### 2.2.1. Influence on the Change of Lee’s Index

**Figure 2:**
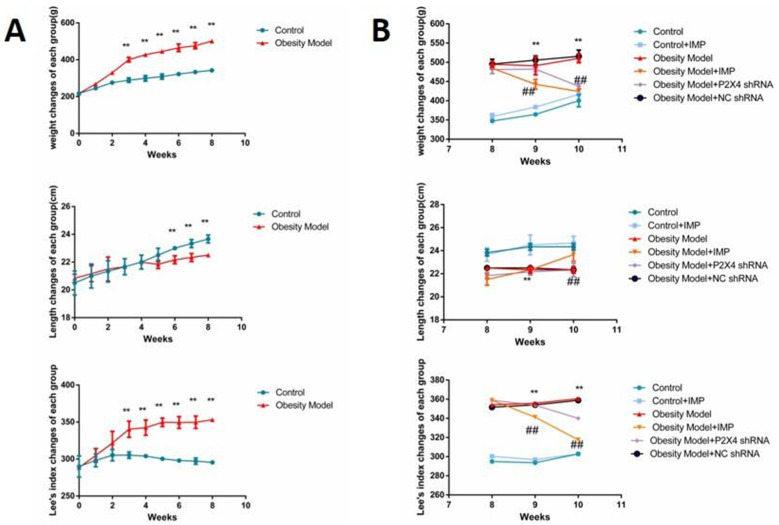
Effect of P2X4 shRNA and imperatorin on the body weight, body length, and Lee’s Index of obese rats. (**A**) The changes in body weight, body length, and Lee’s index of rats in the model group and the control group after high-fat diet. (**B**) The changes in body weight, body length, and Lee’s index of rats in each group after treatment with imperatorin or P2X4 shRNA. *n* = 10 in each group; values are mean ± SD. ** *p* < 0.01 vs. control; ## *p* < 0.01 vs. OM.

Lee’s index is an effective index in evaluating the degree of obesity in adult rats. After feeding with high-fat nutrition balls for 8 weeks, the Lee’s index of the model group was significantly higher than that of the control group (Figure 2A). After treatment, the Lee’s index of rats in the obesity model (OM) + P2X4 shRNA group and the OM + imperatorin group decreased significantly, while the Lee’s index of the OM + NC shRNA group experienced no significant change compared with the OM group (Figure 2B) (*p* < 0.05).

#### 2.2.2. Effects of Imperatorin on Blood Pressure and Heart Rate

The levels of SBP (systolic blood pressure), MBP (mean blood pressure), DBP (diastolic blood pressure), and HR (heart rate) of the OM group were significantly higher than those of the control group. Compared with the OM group, these cardiovascular parameters in obese rats were significantly decreased in the P2X4 shRNA group and imperatorin group after treatment (Table 2). As a negative control, there was no significant change in SBP, MBP, DBP, or HR in the NC shRNA-treated group compared with the OM group (*p* < 0.05).

#### 2.2.3. Effects of Imperatorin on HRV (Heart Rate Variability)

Through frequency domain analysis, it was found that the TP (total power), VLF (very low frequency), LF (low frequency,) and HF (high frequency) of the OM group were decreased significantly, while the LF/HF ratio was clearly increased (*p* < 0.05). After treatment with imperatorin or P2X4 shRNA, the changes in all of these parameters were reversed (*p* < 0.05). There was no significant difference between the OM group and OM + NC shRNA group (Table 3).

#### 2.2.4. Effects of Imperatorin on Sympathetic Activity

**Figure 3:**
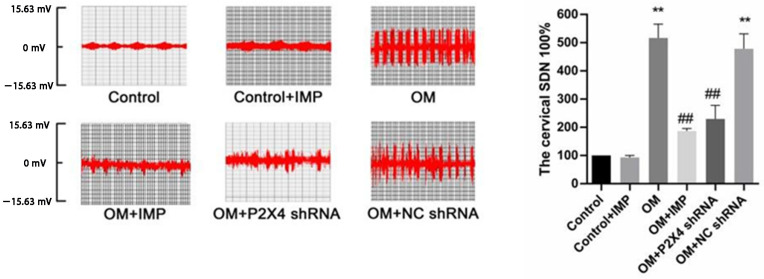
Effects of P2X4 shRNA and imperatorin on sympathetic activity in obese rats. The abnormal discharge of cardiac sympathetic nerve induced by obesity was alleviated after treatment with P2X4 shRNA and imperatorin. *n* = 10 in each group; values are mean ± SD. The experiments were repeated six times independently. ** *p* < 0.01 vs. Control; ## *p* < 0.01 vs. OM.

The sympathetic nerve activity of the obesity group was significantly higher than that of the control group. Compared with the model group, the sympathetic activities of the P2X4 shRNA group and the imperatorin group were significantly decreased after administration (Figure 3) (*p* < 0.05). There was also no significant difference between the ncRNA group and the model group. The results suggest that P2X4 shRNA or imperatorin can alleviate the abnormal excitement of sympathetic activity due to obesity.

#### 2.2.5. Serum Analysis

The contents of triglyceride (TG), total cholesterol (T-CHO), glucose (GLU), and lactate dehydrogenase (LDH-L) in the serum of rats were measured. The results revealed that all of the above indexes of the obesity group were significantly higher than those of the control group, except for GLU, and these changes were decreased after treatment with imperatorin or P2X4 shRNA. There was no significant difference between the OM group and the OM + NC shRNA group (Table 4).

#### 2.2.6. Effect of Imperatorin on the Myocardial Tissue and Tyrosine Hydroxylase (TH) Positive Fibers in the Apex of the Hearts of Obese Rats 

**Figure 4:**
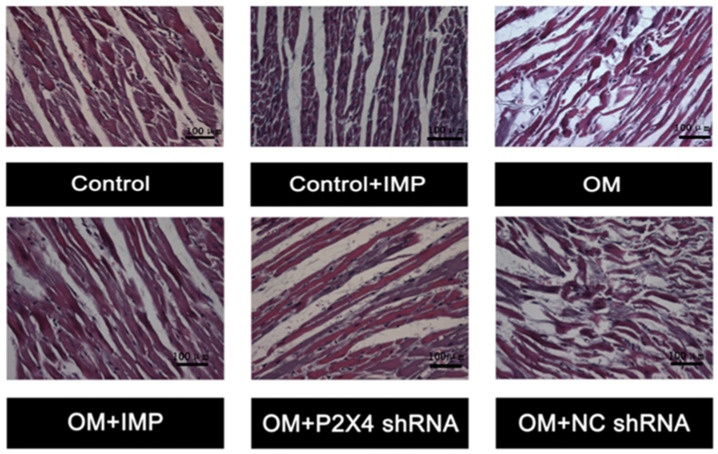
Changes in cardiac muscle structure in the heart apical tissues of rats. Compared with the control group, the muscle organization of the model group was obviously disrupted, which was relieved after treatment with P2X4 shRNA or imperatorin. Magnification of 40×.

The tip part of the hearts of rats was harvested, fixed in 4% paraformaldehyde (4% PFA), and cryosectioned for histology examinations after HE staining (Figure 4). The results showed that compared with the control group, the structure of myocardial tissue in the obese group was significantly disorganized, and the cellular nuclear morphology was abnormal. These anomalous alterations in the myocardial tissue of obese rats were largely prevented by treatments with P2X4 shRNA or imperatorin. 

**Figure 5:**
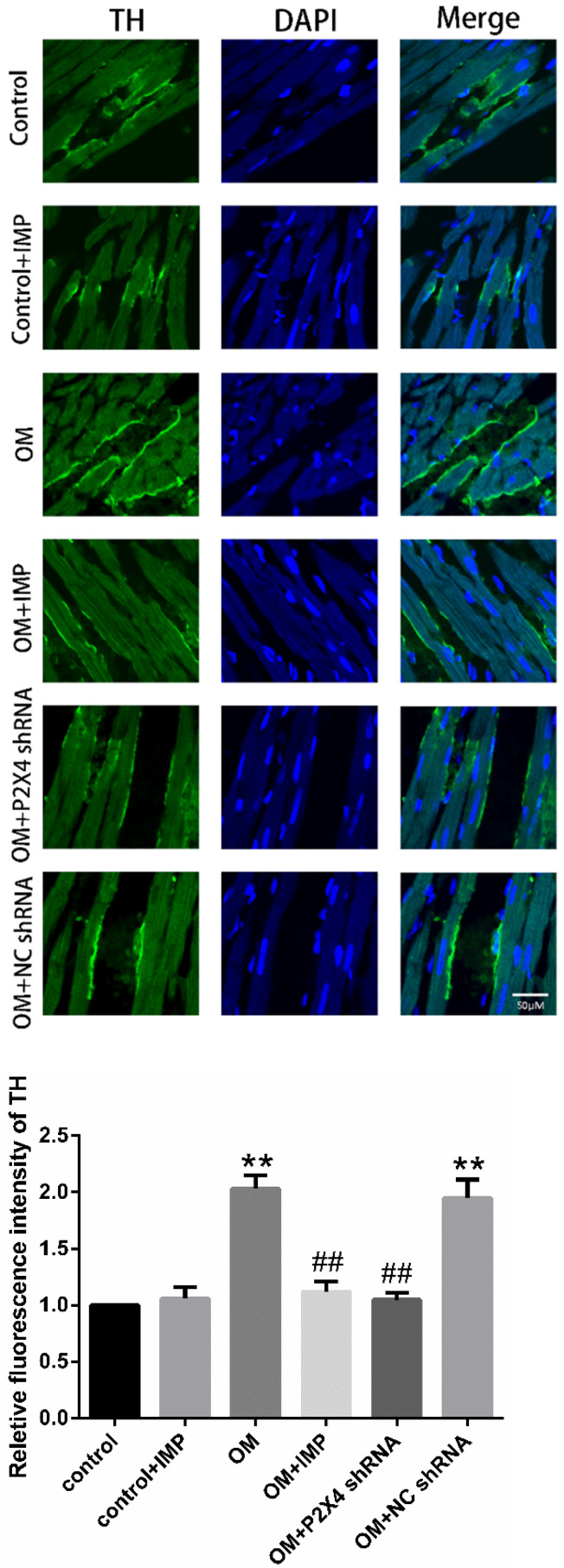
Changes in the intensity of TH-positive fibers in the heart apical tissues of rats. TH was marked with FITC in green light. The bar graph indicates the fluorescence intensity of each group compared to the control group. *n* = 6 in each group; values are mean ± SD. ** *p* < 0.01 vs. control; ## *p* < 0.01 vs. OM. Scale bar = 50 μm.

Changes in the intensity of TH-positive fibers in the heart apical tissues of rats were observed using immunofluorescence staining. The results showed that the fluorescence intensity of TH-positive fibers was increased in the OM group compared to the control group (*p* < 0.01), while treatment with P2X4 shRNA or imperatorin reduced the increased expression (*p* < 0.01) (Figure 5). 

### 2.3. Effect of Imperatorin on the Expression of P2X4 in the SG of Obese Rats

**Figure 6:**
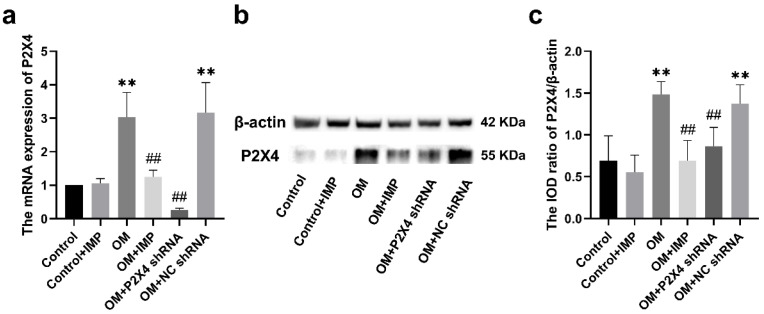
Effect of imperatorin on the expression of P2X4 in the SG of obese rats. Changes in mRNA (**a**) and protein levels (**b**,**c**) of P2X4 in the SG of rats were detected via qPCR and Western blotting, respectively. *n* = 6 in each group; values are mean ± SD. ** *p* < 0.01 vs. Control; ## *p* < 0.01 vs. OM.

Real-time quantitative PCR showed that P2X4 expression at the mRNA level in the OM group was significantly higher than that in the control group (*p* < 0.01), whereas after treatment with P2X4 shRNA or imperatorin, the P2X4 expression level was significantly lower compared to the OM group (*p* < 0.01) (Figure 6a).

Western blotting analysis showed that P2X4 (Figure 6b,c) protein content was significantly higher in the OM group than in the control group (*p* < 0.05). The expression levels of the P2X4 protein in the OM + P2X4 shRNA and OM + imperatorin groups were significantly lower than that in the OM group.

**Figure 7:**
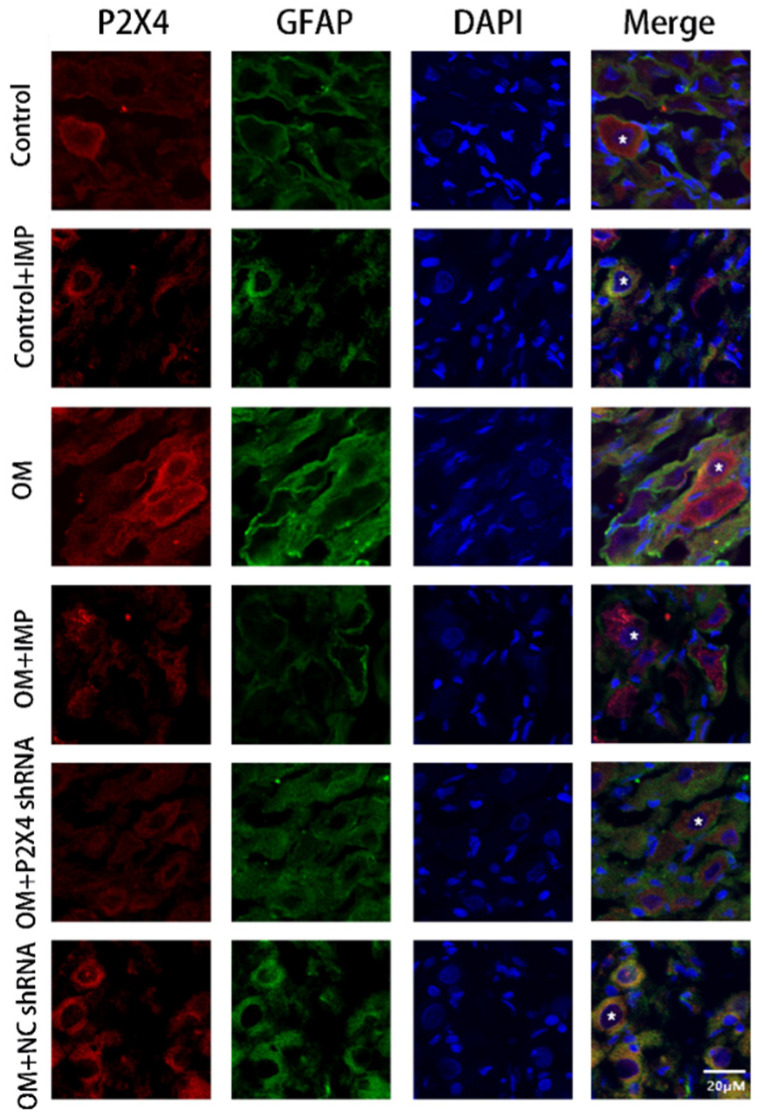

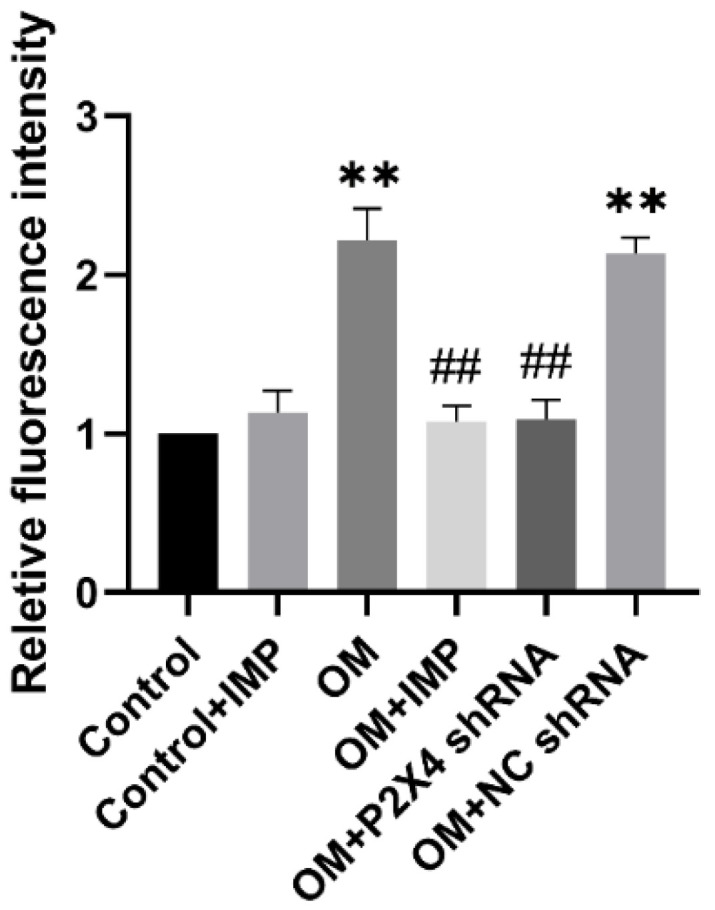
Immunofluorescence double-labeling of the co-expression of P2X4 and GFAP in the SG of obese rats, as assessed under a confocal fluorescence microscope. P2X4 was marked with TRITC in red light, and GFAP with FITC in green light. The combined images showed the co-expression level of P2X4 and GFAP. The bar graph indicates the fluorescence intensity of co-expression in each group compared to the control group. *n* = 6 in each group; values are mean ± SD. ** *p* < 0.01 vs. control; ## *p* < 0.01 vs. OM. * indicates location of neuronal cell body. Scale bar = 20 μm.

The co-expression of P2X4 receptors with glial fibrillary acidic protein (GFAP, a satellite glial cell marker) was assessed using the immunofluorescence double-labeling technique (Figure 7). SGCs are the major glial cells in the sympathetic ganglia, and they specifically contact neuronal cell bodies. The results of GFAP fluorescence staining showed that in the stellate sympathetic ganglion, SGCs surround the single neuronal cell body and form a ring-like structure, which was similar to that reported by other researchers [21]. The assay validated the co-expression of P2X4 receptors with GFAP in the SG, suggesting that P2X4 is predominantly present in satellite glial cells. The results revealed that the extent of co-expression of P2X4 with GFAP in the SG was increased in the OM group compared to the control group (*p* < 0.01), while treatment with P2X4 shRNA or imperatorin reduced such upregulated co-expression (*p* < 0.01). 

### 2.4. Effect of Imperatorin on Inflammatory Factor Production in Obese Rats

**Figure 8:**
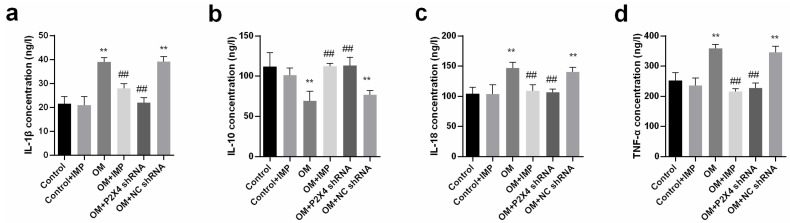
Effects of P2X4 shRNA and imperatorin on the changes in inflammatory factors in the serum of obese rats. The bar charts show the contents of IL-1β (**a**), IL-10 (**b**), IL-18 (**c**), and TNF-α (**d**) in serum of rats under various experimental conditions. *n* = 6 in each group; values are mean ± SD. ** *p* < 0.01 vs. Control; ## *p* < 0.01 vs. OM.

The ELISA results showed that the serum levels of IL-1β (Figure 8a), TNF-α (Figure 8d), and IL-18 (Figure 8c) in the OM group were significantly higher than that in the control group (*p* < 0.05), while treatment with P2X4 shRNA or imperatorin significantly reduced the levels of the above inflammatory factors. No significant difference was seen between the OM + ncRNA group and the OM group (*p* > 0.05). In contrast, the serum IL-10 level was significantly lower in the OM group compared to the control group (*p* < 0.05). P2X4 shRNA and imperatorin treatment could elevate IL-10 in obese rats (*p* < 0.05) (Figure 8b).

**Figure 9:**
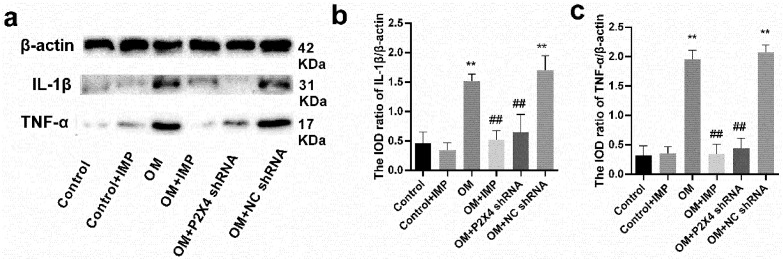
Changes in IL-1β and TNF-α protein expression in the SG of obese rats by imperatorin via Western blotting (**a**). The bar graphs show statistical analysis of the integrated optical density (IOD) ratio for IL-1β (**b**) and TNF-α (**c**) relative to β-actin. *n* = 6 in each group; values are mean ± SD. ** *p* < 0.01 vs. control; ## *p* < 0.01 vs. OM.

The changes in the inflammatory factors IL-1β and TNF-α in rat SG were also examined via Western blotting (Figure 9). The results showed that the expression of IL-1β and TNF-α in the OM group (*p* < 0.01) were significantly higher than those in the control group. However, the expression levels of IL-1β and TNF-α in obese rats were significantly lower (*p* < 0.01) after P2X4 shRNA and imperatorin treatment. There was no significant difference between the OM group and the OM + NC shRNA-treated group (*p* > 0.05).

### 2.5. Effect of Imperatorin on Cellular Pyroptosis in SG

**Figure 10:**
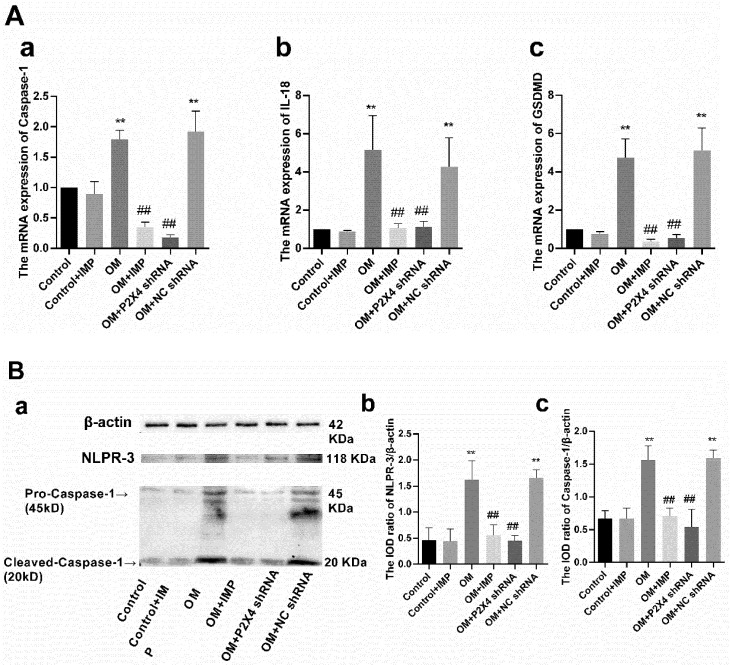
(**A**) Effects of P2X4 shRNA and imperatorin on the up-regulated expression of mRNA of caspase-1 (**a**), IL-18 (**b**), and GSDMD (**c**) in the SG of obese rats, as detected by RT-qPCR. (**B**) Effects of P2X4 shRNA and imperatorin on the expression of NLRP-3 and caspase-1 in SG of obese rats, assessed using Western blotting (**a**). Bar graphs (**b**,**c**) show the statistical results of the integrated optical density (IOD) ratio of caspase-1 and NLRP-3, respectively, relative to β-actin. *n* = 6 in each group; values are mean ± SD. ** *p* < 0.01 vs. Control; ## *p* < 0.01 vs. OM.

To explore whether the pyroptosis mediates obesity-triggered sympathetic neuropathy, the expression levels of certain relevant key players were investigated. qPCR results showed that the mRNA levels of caspase-1, IL-18, and GSDMD were significantly higher in the SG of OM rats than in the control group (Figure 10A), (*p* < 0.01); after treatment with P2X4 shRNA or imperatorin, the mRNA levels of all these molecules were significantly decreased (*p* < 0.05). The changes at the protein level of caspase-1 and NLRP-3 were detected via Western blotting (Figure 10B), which revealed that the protein contents of caspase-1 and NLRP-3 in the SG of the OM group were significantly higher than those of the control group (*p* < 0.01). Moreover, treatment with P2X4 shRNA or imperatorin could significantly decrease the expression of caspase-1 and NLRP-3 in obese rats (*p* < 0.01).

### 2.6. Effect of Imperatorin on ERK1/2 Phosphorylation in MAPK Family in SG

**Figure 11:**
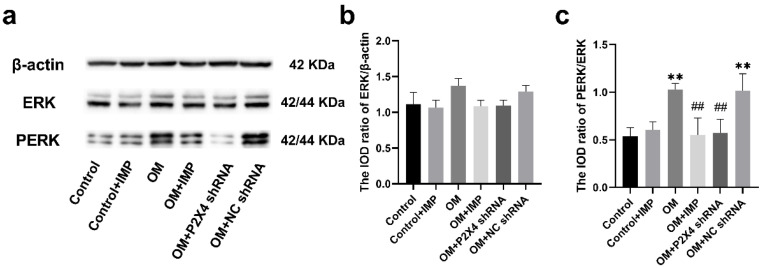
Effects of imperatorin on the expression of ERK1/2 and P-ERK1/2 in the SG of obese rats, as assessed using WB (**a**). Bar graphs (**b**,**c**) show the statistical results of the integrated optical density (IOD) ratio of ERK1/2 and P-ERK1/2, respectively, relative to β-actin and ERK1/2. *n* = 6 in each group; values are mean ± SD. ** *p* < 0.01 vs. Control; ## *p* < 0.01 vs. OM.

Western blotting was used to determine the expression changes of the MAPK family proteins (ERK1/2 and its active form P-ERK1/2) in the SG of rats in each group (Figure 11). The results showed that there was no significant difference in the expression of ERK1/2 in the SG of any group (*p* > 0.05). However, the expression of the phosphorylated P-ERK1/2 differed significantly between the groups: it was significantly higher in the OM group compared to the control group (*p* < 0.01) and was highly inhibited after treatment with imperatorin or P2X4 shRNA (*p* < 0.01).

### 2.7. Effect of Imperatorin on ATP-Activated Current in HEK293 Cells Transfected with pEGFP-hP2X4 Plasmid

**Figure 12:**
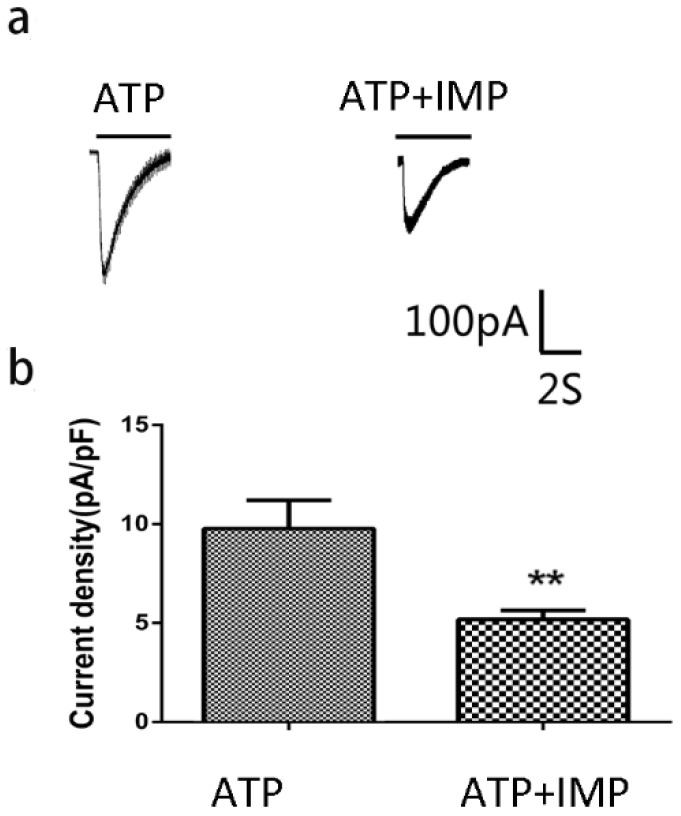
Effects of imperatorin on ATP-activated current in HEK293 cells transfected with hP2X4 receptor. (**a**) Original tracings show the ATP (100 μM)-induced currents in HEK293 cells transfected with the hP2X4 receptor, and IMP (100 μM) inhibited the ATP-induced currents. (**b**) The histogram showed the statistical analysis of current density with or without IMP. *n* = 6 in each group; values are mean ± SD. ** *p* <0.01 vs. ATP alone.

We used the whole-cell patch clamp technique to determine the effects of IMP on ATP-activated currents in HEK293 cells transfected with pEGFP-hP2X4 plasmid. We found that P2X4 receptor-expressing HEK293 cells were activated by 100 μM ATP. Moreover, IMP (100 μM) significantly inhibited the ATP-activated current (*p* < 0.01; Figure 12).

## 3. Discussion

When the body’s energy intake exceeds its expenditure, the excess energy is stored in adipose tissue, leading to obesity. Adipocytes are important inflammatory cells in the body that can secrete many inflammatory factors, such as tumor necrosis factor alpha (TNF-α) and interleukins (IL), which interfere with insulin signaling through the bloodstream or by paracrine action, leading to IR [22,23]. During obesity, adipose tissue also secretes angiopoietin-like protein 2 or leptin, leading to increased inflammation and the development of cardiovascular diseases [24]. Obesity can cause changes in the structure and function of the heart, and thus increase the occurrence of serious cardiovascular diseases such as heart failure and sudden cardiac death [25]. Obesity induces pathological alterations in the heart-related renin-angiotensin-aldosterone system, which increases the secretion of aldosterone and promotes myocardial fibrosis, platelet aggregation, and endothelial dysfunction, leading to cardiomyocyte hypertrophy and apoptosis [26].

The cardiac autonomic neuropathy can be triggered by obesity and be improved by regular physical activity and diet-induced weight loss [27]. In this study, the Lee’s index, blood pressure, heart rate variability, sympathetic discharge, and serum levels of TG, T-CHO, GLU, and LDH-L were measured after rats were fed high-fat foods for 8 weeks. The results showed that these obese rats displayed significantly higher Lee’s index and blood pressure values. Furthermore, we observed changes in the autonomic nerve function of obese rats. The heart rate variability was altered in obese rats, including reduced TP (Total power), VLF (Very low frequency), LF (low frequency), and HF (High frequency), as well as significantly increased LF/HF ratio. Post-ganglionic nerve fibers of the SG run to the cardiac plexus, which can affect the sympathetic efferent activity of the heart and hence play an important role in regulating heart rate, myocardial contraction, and relaxation. The SG is usually a fusion of the seventh cervical ganglia and the first thoracic ganglion [28]. Thus, in this study, we furthermore observed the cervical sympathetic nerve activity and TH fluorescence intensity in the heart. The obese rats also showed an abnormal increase in sympathetic nerve discharge and TH fluorescence intensity in the heart. Moreover, serum analysis revealed significantly higher lipids in obese rats. It is known that obesity plays a key role in the development of insulin resistance, since the degree of IR is correlated with the degree of obesity. However, in this study the rats were always in the normal blood glucose range, indicating that they did not yet develop diabetes. This is consistent with observations made in previous studies [29]. All these results demonstratively identify obesity as a cause of cardiac autonomic neuropathy, which is in agreement with the notion posed in a previous study [25]. 

The P2X4 isoform is widely expressed in the nervous system for sensing extracellular ATP stimulation and may be involved in both neuronal excitatory and inhibitory signaling as a modulator [30,31]. The SG participates in cardiac sympathetic efferent activity [32], and there are many P2X4 receptors on the satellite glial cells of the SG [9,10]. In this study, after the development of cardiac autonomic nervous lesions in the obesity model of rats, we treated obese rats with imperatorin by gavage for 2 weeks or with sublingual intravenous injections of the P2X4 shRNA plasmid. We found that imperatorin or P2X4 shRNA treatment had mitigating effects on the degree of obesity, abnormalities in blood pressure, increases in heart rate variability, abnormal cardiac sympathetic nerve activity, and the elevation of serum TG, T-CHO, and LDH-L levels in obese rats. In addition, both Western blotting and qPCR showed significantly higher expression of P2X4 receptors in the SG of obese rats compared to controls. The results of immunofluorescence indicated that the co-location of P2X4 with GFAP was significantly higher in the SGs of the obese rat model group, suggesting that the elevated expression of P2X4 receptors in the SGs of obese rats was mainly on satellite glial cells. The up-regulated P2X4 receptors in the SGs of obese rats were significantly reduced after treatment with imperatorin or P2X4 shRNA interference. Since the interference efficacies of imperatorin and P2X4 shRNA treatment were similar, the mechanism of action for imperatorin in protecting against obesity-induced cardiac sympathetic neuropathic injury should be closely related to the downregulation of P2X4 receptors on satellite glial cells. Furthermore, molecular docking results showed that the P2X4 receptor had good binding ability with imperatorin. We used the whole-cell patch clamp technique to determine the effects of IMP on ATP-activated currents in HEK293 cells transfected with pEGFP-hP2X4 plasmid. We found that IMP significantly inhibited the ATP-activated current. Therefore, we speculated that the up-regulated P2X4 receptors in the SGs of obese rats might mediate the obesity-induced abnormal cardiac sympathetic nerves injury, and the protective effect of imperatorin may be related to inhibiting the expression of P2X4 receptors of the SG.

Obesity may trigger cardiac sympathetic neuropathy under a state of systemic hypo-inflammatory response [23]. It has been reported that the levels of pro-inflammatory TNF-α and IL-1β are usually obviously increased in obese rats [33]. However, IL-10 acts as an anti-inflammatory factor in the immune response related to obesity [34]. Similar results were seen in this study, i.e., significantly increased serum levels of TNF-α and IL-1β alongside significantly decreased IL-10 levels in obese rat serum. Moreover, these changes could be reversed after treatment with imperatorin or P2X4 shRNA. Western blot results also confirmed that the production of IL-1β and TNF-α was significantly up-regulated in the SG, and the events were significantly decreased by imperatorin or P2X4 shRNA administration. These findings suggest that inflammatory factors are closely associated with obesity-induced cardiac sympathetic neuropathy, and the protective effect of imperatorin may be attributable to its anti-inflammatory ability.

Pyroptosis is a kind of programmed cell death related to caspase-1 activation that triggers the release of several proinflammatory factors such as TNFα, IL-1β, and IL-18 [35]. Caspase-1, NLRP-3, and GSDMD are key factors linked to pyroptosis [36,37]. The qPCR results showed that the mRNA levels of caspase-1, IL-18, and GSDMD were all elevated in the SG of obese rats compared to the control group. Similar results were obtained through Western blotting, which revealed significant increases in the protein contents of caspase-1 and NLRP-3 in the SG of obese rats. The measurements from ELISA also showed that the serum levels of TNFα, IL-1β, and IL-18 in the obese group were significantly higher than those in the control. All of the above pathological changes could be improved after treatment with imperatorin or P2X4 shRNA. Our previous work has demonstrated that the increased P2X4 receptors in DRG may induce pyroptosis in gp120-induced neuronal injury [18]. These results together suggest that imperatorin may ameliorate obesity-induced cardiac sympathetic neuropathy in rats, resulting from the development of an inflammatory state which can trigger the activation of the NLRP3/caspase-1/GSDMD-implicated pyroptosis pathway via the mediation of increased expression of P2X4 receptors in the SG. P2X4 receptors are not only expressed on the glial cells but are also expressed on other cells, such as CD4+ T-cells, macrophages, and adipocytes [38,39]. Whether P2X4 receptors expressed on other cells contribute to release of proinflammatory cytokines and pyroptosis, or affect the sympathetic nerve activity is unclear and requires more investigation in future. Likewise, the possibility that IMP and P2X4 shRNA also act on these cells to exert effects cannot be ruled out at the moment.

The MAPK pathway may participate in the CNS response to challenges in the surrounding environment, and the ERK-MAPK signaling pathway in the CNS is closely linked to the P2X4 receptor [40]. Activation of P2X4 receptor may cause activation of the ERK pathway, which is also tightly related to the synthesis of inflammatory factors [41]. In the present study, we found that the activity of the ERK-MAPK pathway was significantly increased in the SG of obese rats, as indicated by the elevated expression of P-ERK1/2. On the contrary, treatment with imperatorin or P2X4 shRNA interference reduced the activation of the ERK-MAPK pathway in obese rats. These data suggest that the inhibition of the ERK pathway activation may contribute to the molecular mechanism for the protective effect of imperatorin on obesity-induced cardiac sympathetic nerve injury.

## 4. Materials and Methods

### 4.1. Experimental Animals

Seven-week-old SD male rats (200 ± 20 g) were provided by the Changsha Tianqin Biological Corporation, Changsha, China. The animal experiment was approved by the Animal Ethics Committee of the Medical College of Nanchang University.

### 4.2. Establishment of Obese Rat Model

**Figure 13:**
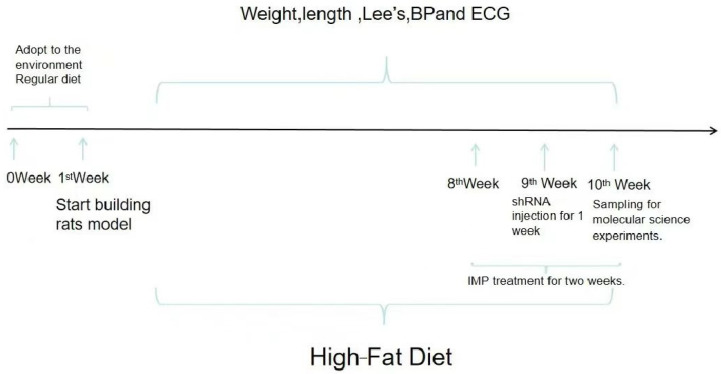
The timeline of the model establishment and sample collection.

All rats were placed in a suitable environment and given a normal diet and adequate water supply. After adapting to the environment for a week, the rats were randomly divided into a control group and an obesity model group. The control group continued to receive a normal diet. Model rats were fed with high-fat nutrition balls (73% basic feed, 10% lard, 10% sucrose, 5% egg yolk powder, 1.5% cholesterol, and 0.5% bile salt) for 8 weeks [42]. After 8 weeks, the rats’ Lee’s index (Lee’s = $\sqrt[{3}]{\mathrm{body}\;\mathrm{weight}}$/body length × 100), blood pressure, heart rate, systolic blood pressure, diastolic blood pressure, and mean arterial pressure were measured. The Lee’s index was 10% higher than normal rats, and the modelling criteria included systolic blood pressure ≥ 140 mmHg, diastolic blood pressure ≥ 90 mmHg and mean arterial pressure ≥ 100 mmHg. In total, 80 suckling rats were used to establish the obesity model group. After modeling, the rats in the model group were randomly divided into four groups: obesity model group (OM group), obesity model + P2X4 short hairpin RNA group (OM + P2X4 shRNA group), obesity model + nc short hairpin RNA group (OM + NC shRNA group), and obesity + imperatorin group (OM + IMP group). The timeline of the model establishment and sample collection was showed in Figure 13.

The transfection complex, consisting of shRNA (P2X4 shRNA or NC shRNA) and transfection reagent at a ratio of 1:2 (μg/μL), was prepared using an Entranster™ in vivo transfection kit (Engreen Biosystem Company of Beijing), according to the manufacturer’s instructions. The P2X4 shRNA sequences were as follows:

GATCCCGTCTACTGCATGAAGAAGAATTGATAT

CCGTTCTTCTTCATGCAGTAGATTTTTTCCAAA

During the 8th week, P2X4 shRNA and NC shRNA were injected into the OM + P2X4 shRNA group and OM + NC shRNA group through the sublingual vein at a dose of 5 µg/kg for one week, once every other day. The imperatorin (Sichuan Chengdu Phytolabeled Chemical Pure Biotechnology Company, Chengdu, China) was dissolved in 0.5% methyl cellulose sodium and added to double distilled water. The rats in the relevant group were intragastrically administrated with the imperatorin at a dose of 100 mg/kg for 2 weeks, once per day [43].

### 4.3. Molecular Docking 

Molecular docking predicts affinity by studying the interaction between small ligand molecules and receptor biomacromolecules [44]. The binding ability between imperatorin and the P2X4 receptor was predicted using a semi-flexible docking method. The molecular docking of imperatorin and P2X4 was carried out via homologous modeling [45]. The P2X4.pdb file containing the P2X4 receptor protein sequences was downloaded from http://www.rcsb.org/pdb/home/home.do (accessed on 12 February 2022), and the imperatorin.sdf file was downloaded from https://pubchem.ncbi.nlm.nih.gov/ (accessed on 12 February 2022). After pretreating with pyMOL software (The PyMOL Molecular Graphics System, Version 2.0 Schrödinger, LLC.) to remove small molecule ligands, dehydrate, and hydrogenate, autodock tools software (La Jolla, CA, USA) was applied for molecular docking based on python.

### 4.4. Noninvasive Blood Pressure Measurement of Caudal Artery in Rats

The rats were placed in a rat bag to avoid light, and after it was quiet, the pressure sensor was fixed on one of three points on the tails of rats. The heart rate (HR), systolic blood pressure (SBP), mean arterial pressure (MBP), and diastolic blood pressure (DBP) were measured using the indirect tail sleeve method [46]. This was repeated on each rat three times, and the average value was taken as the test result. All measurements were blind to the evaluators.

### 4.5. Heart Rate Variability (HRV)

Heart rate variability (HRV) refers to the slight variation between successive sinus cardiac cycles, which can reflect the functional state of the cardiac autonomic nervous system [47]. The second lead electrocardiograph (ECG) was recorded using the Medlab biological signal acquisition and processing system, and the heart rate variability was analyzed by short-term 5-min frequency domain analysis. The parameters of HRV analysis include total power (TP) (0–0.5 Hz), very low frequency (VLF) (0.003–0.04 Hz), low frequency (LF) (0.04–0.15 Hz), and high frequency (HF) (0.15–0.40 Hz). LF represents sympathetic activity, HF represents vagus activity, and LF/HF ration represents the balance of sympathetic and vagal activity. All measurements were blind to the evaluators.

### 4.6. Recording the Electrical Activity of Cervical Sympathetic Nerve

After general anesthesia, rats were fixed on the anatomical plate in the supine position, the left cervical sympathetic ganglion was dissected, and the postganglionic sympathetic nerve fibers were bluntly separated. The isolated sympathetic nerve was suspended on the physiological gold electrode, and the nerve was wrapped with paraffin oil to isolate and moisten the nerve. The black lead was clamped on the thigh skin, and the other end was connected to the RM6240 biological function experiment system to record the sympathetic nerve discharge [46]. 

### 4.7. Serum Analysis

The blood samples were stored at room temperature for 2 h, followed by centrifugation at 3000 rpm for 15 min. The supernatant was taken for packaging, and the samples were stored at −80 ℃ to avoid repeated freeze-thaw. The reagents were prepared, and the measurements were conducted according to the instructions by automatic detection instrument.

### 4.8. HE Staining

Sections of 10-μm from the specimens were prepared with myocardium stored in PFA. After splicing the wax blocks containing the rectums, the specimens were kept at 37 °C overnight. Then, the specimens were baked in an oven at 60 °C for 2–3 h before two rounds of dewaxing in xylene I and then xylene II. Subsequently, the specimens went through a 5 min hydration in 100, 95, 75, and 50% ethanol in serial order, followed by 5 min of washing with PBS. Thereafter, the specimens were dyed with hematoxylin, which was followed by 5 min of rinsing under running water. Then, the specimens were differentiated with 1% hydrochloric acid alcohol before another 1 h rinse under running water. Eventually, the specimens were colored for 5 min using eosin, dehydrated, and sealed using neutral resin.

The HE staining of myocardium of rats in each group was observed, the pathological changes of myocardium were observed under light microscope [48].

### 4.9. Western Blot

Western blot was used to detect the protein expression changes of P2X4, P-ERK1/2, IL-1β, TNF-α, caspase-1, and NLRP-3 using the antibodies with dilution ratio 1:800, 1:1000, 1:200, 1:800, 1:800, and 1:500 respectively. SGs preserved at −80 were lysed by 98% RIPA lysate, 1% protease inhibitor, and 1% protein phosphatase inhibitor after grinding in a homogenizer. After being centrifuged at 4° and 12,000 rpm for 10 min, the supernatant was aspirated and denatured by adding six times the loading buffer at a ratio of 5:1 and kept in boiling water for 5 min. Extracted proteins were loaded on 10% sodium dodecyl sulfate–polyacrylamide gel for electrophoresis with a Bio-Rad system. The protein samples in each well were 5–10 μg. The protein bands were transferred onto polyvinylidene fluoride membranes (PVDF membranes), which were then blocked with 5% skim milk in 1× TBST (Cwbio, Beijing, China) for 2 h. After a gentle rinse, the membrane was immersed in the primary antibodies against β-actin (ZSGB-Bio, Beijing, China), the P2X4 receptor (Cat.APR-002, Alomone, Jerusalem, Israel), extracellular signal–regulated kinases 1/2 (ERK 1/2) (Cell Signaling Technology, Danvers, MA, USA), and phosphorylated (p)-ERK1/2 (Cell Signaling Technology, Danvers, MA, USA) overnight at 4 ℃. Thereafter, the membrane was incubated with a second antibody: horseradish peroxidase–conjugated goat anti-rabbit IgG (ZSGB-Bio, Beijing, China) or goat anti-mouse IgG (ZSGB-Bio, Beijing, China) in blocking buffer. Subsequently, the PVDF membrane was treated with 1:1 diluted ECL luminous solution (Advansta, Menlo Park, CA, USA) and exposed in a gel imaging system (BIO-RAD, Hercules, CA, USA). The images were analyzed by ImageJ system, which calibrated the IOD values of target protein bands and the corresponding β-actin bands. Eventually, the optical density ratio of target protein to β-actin was calculated.

### 4.10. Quantitative Real-Time PCR

Under the condition of no ribozyme, the RNA of the SG was extracted, immersed in RNA store solution, and stored in −80 °C. The full-type gold kit (Transgen, Beijing, China) was used for RNA reverse transcription. The primer sequences are as follows: β-actin forward TGT CAC CAA CTG GGA CGA TA, reverse GGG GTG TTG AAG GTC TCA AA; P2X4 forward AAG GTG TGG CTG TGA CCA AC, reverse AGG AAT CTC TGG ACA GGT GC; caspase-1 forward GGAGCTTCAGTCAGGTCCAT, reverse GCGCCACCTTCTTTGTTCAG; GSDMD forward GCCAGAAGAAGACGGTCACCATC, reverse TTCGCTCGTGGAACGCTTGTG; and IL-18 forward GACTGGCTGTGACCCTATCT, reverse TTCCATTTTGTTGTGTCCTG.

Quantitative real-time PCR was conducted using StepOne (ABI, Thermo Fisher Scientific, CA, USA). Each independent sample was tested three times to obtain the average value [49]. The ΔΔCT method was used to quantify the expression of each gene, with CT as the threshold cycle. The relative levels of target genes normalized to the individual sample with the lowest CT are presented as 2^−ΔΔCT^.

### 4.11. Immunofluorescence Labeling

The immunofluorescence double labeling technique was used to observe the co-expression of P2X4 and glial fibrillary acidic protein (GFAP) (a satellite glial cell marker) and to detect changes in the level of P2X4 in satellite glial cells of the SG in each group. The SGs were taken out and underwent 4% paraformaldehyde fixation. The cryosectioned samples were washed using PBS for three rounds of 5 min each before undergoing 1 h of blocking with gout serum at 37 ℃. Thereafter, the specimens were incubated in a mixed antibody solution containing both anti-P2X4 (1:100) (Alomone, Jerusalem, Israel) and anti-GFAP (1:100) (Invitrogen, Carlsbad, CA, USA) at 4 °C overnight. Subsequently, the specimens were incubated with goat anti-rabbit tetramethylrhodamine (TRITC) 1:200 (Thermo Fisher Scientific, Carlsbad, CA, USA) and goat anti-mouse fluorescein isothiocyanate (FITC) 1:200 (Thermo Fisher Scientific, Carlsbad, CA, USA). Then, the specimens were stained with 4, 6-diamidino-2-phenylindole (DAPI) for 5 min and sealed with an anti-fluorescence attenuation agent, before examination under a confocal fluorescence microscope (Olympus, FV3000, Tokyo, Japan). The mean optical density value was calculated by ImageJ software and used to describe the staining intensity [49].

Immunofluorescence labeling technique was also used to observe the expression of tyrosine hydroxylase (TH) and to detect changes in the level of TH in the apex of the hearts in each group. Anti-TH (1:100) (Proteintech, Wuhan, China) and goat anti-rabbit FITC (1:200) (Abcam, Cambridge, UK) were used with the same protocol as above.

### 4.12. Enzyme-Linked Immunosorbent Assay (ELISA) 

ELISA kits (Boster Biological Technology, Wuhan, China) were used to detect the levels of IL-1β, TNF-α, IL-10, and IL-18 in rat serum. The absorbance (OD) was measured using a multi-function microplate reader (PerkinElmer, Waltham, MA, USA) at 450 nm wavelength, and the levels of targeted molecules in the serum were calculated using a respective standard curve.

### 4.13. Whole-Cell Electrophysiological Recordings 

Electrophysiological recording was carried out via a patch/whole-cell clamp amplifier (Axopatch 200B). HEK293 cells were transfected with the human pcDNA3.0-EGFP- P2X4 plasmid using Lipofectamine 2000 reagent (Invitrogen) according to the manufacturer’s instructions. Whole-cell patch clamp recordings were carried out 2 days after transfection. HEK293 cells that expressed green fluorescence were subjected to electrophysiological recording [50]. The cells were divided randomly into 2 groups: one for ATP administration alone, another for incubation with imperatorin for 1 min followed by ATP administration.

### 4.14. Statistical Analysis

SPSS26 software (IBM, New York, NY, USA) was used for statistical analysis. The data were expressed as mean ± standard deviation (SD). The statistical analysis chart was made by GraphPad Prism7 (8.0.2, GraphPad software, San Diego, CA, USA). Data were analyzed via one-way analysis of variance (ANOVA) followed by the LSD post-hoc test for multiple comparisons. *p* < 0.05 was considered to indicate a significant difference.

## 5. Conclusions

Upregulated P2X4 in the SG is involved in the pathological process of obesity-induced cardiac sympathetic neuropathy, inflammation, and abnormal cardiovascular function. Imperatorin can protect against P2X4 receptor-mediated, obesity-induced cardiac sympathetic neuropathy in rats by inhibiting the activation and expression of P2X4 receptors; the action of imperatorin may be attributable to the inhibition of cellular pyroptosis, reduction of inflammatory factor levels, and inhibition of ERK pathway activation. Thus, imperatorin could be an effective potential compound for the management of obesity-induced cardiac sympathetic neuropathy.

## Figures and Tables

**Table 1:** Molecular docking data sheet for imperatorin and P2X4 receptors.

Mode	Affinity	Dist. from Best Mode	
	(kcal/mol)	rmsd l.b.	rmsd u.b.
1	−7	0	0
2	−6.6	1.301	1.723
3	−6.3	21.545	22.744
4	−6.3	1.169	1.553
5	−6.2	20.781	22.502
6	−6	22.813	24.75
7	−5.9	20.806	22.573
8	−5.8	23.623	25.804
9	−5.8	23.077	25.345

The predicted binding affinity is displayed in kcal/mol (energy). rmsd: RMSD values were calculated relative to the best mode and only used movable heavy atoms. Two variants of RMSD metrics are provided: rmsd (RMSD lower bound: matches each atom in one conformation with itself in the other conformation, ignoring any symmetry) and rmsd/ub (RMSD upper bound: rmsd/lb [c1,c2] = max [rmsd’{c1,c2}, rmsd’{c2,c1}]; and rmsd’ matches each atom in one conformation with the closest atom of the same element type in the other conformation), which differ in how their atoms are matched in the distance calculation. There was a strong reaction between ligands and proteins, and the molecular docking of imperatorin to P2X4 was stable.

**Table 2:** Effects of P2X4 shRNA and imperatorin (IMP) on heart rate and blood pressure in obese rats.

	HR (Times/Minute)	SBP (mmHg)	MBP (mmHg)	DBP (mmHg)
Control	379 ± 8.025	113.833 ± 7.111	86.167 ± 6.178	73.167 ± 7.935
Control + IMP	394.167 ± 31.141	113.667 ± 5.428	83.333 ± 7.23	73.333 ± 4.546
OM	487 ± 22.512 **	154.833 ± 7.139 **	114.333 ± 7.763 **	110.167 ± 3.125 **
OM + IMP	345.333 ± 28.759 ^##^	112 ± 8.295 ^##^	86.167 ± 6.432 ^##^	80.167 ± 5.037 ^##^
OM + P2X4 shRNA	354.333 ± 46.779 ^##^	116.167 ± 5.672 ^##^	87 ± 9.033 ^##^	80.667 ± 9.852 ^##^
OM + NC shRNA	487.167 ± 9.196	148.333 ± 9.092	110.667 ± 6.25	103.5 ± 3.332

*n* = 10 in each group; values are mean ± SD. ** *p* < 0.01 vs. Control; ^##^ *p* < 0.01 vs. OM.

**Table 3:** Effects of P2X4 shRNA and imperatorin on HRV.

	VLF (ms^2^)	LF (ms^2^)	HF (ms^2^)	TP (ms^2^)	LF/HF
Control	705.157 ± 54.058	512.930 ± 42.453	452.715 ± 35.336	1730.083 ± 18.640	1.368 ± 0.226
Control + IMP	695.585 ± 47.050	488.102 ± 55.513	432.888 ± 18.790	1893.083 ± 372.618	1.372 ± 0.173
OM	150.298 ± 3.116 **	207.722 ± 2.998 **	95.513 ± 5.888 **	616.023 ± 72.410 **	2.365 ± 0.095 **
OM + IMP	238.738 ± 16.847 ^##^	765.258 ± 38.066 ^##^	634.445 ± 68.720 ^##^	840.737 ± 86.507 ^##^	1.220 ± 0.175 ^##^
OM + P2X4 shRNA	514.635 ± 20.028 ^##^	628.565 ± 14.912 ^##^	571.692 ± 10.876 ^##^	3060.778 ± 42.437 ^##^	1.100 ± 0.014 ^##^
OM + NC shRNA	138.635 ± 3.504	208.465 ± 2.681	83.140 ± 2.101	498.698 ± 117.110	2.510 ± 0.087

Treatment with P2X4 shRNA and imperatorin reversed the obesity-induced changes in HRV. *n* = 10 in each group; values are mean ± SD. The experiments were repeated six times independently. ** *p* < 0.01 vs. Control; ^##^ *p* < 0.01 vs. OM.

**Table 4:** Effects of P2X4 shRNA and imperatorin on serum lipids and glucose.

	TG (mmol/L)	T-CHO (mmol/L)	LDH-L (mmol/L)	GLU (mmol/L)
Control	0.843 ± 0.218	1.807 ± 0.066	739.990 ± 154.867	8.967 ± 3.219
Control + IMP	0.813 ± 0.248	1.655 ± 0.144	750.889 ± 119.992	9.967 ± 2.799
OM	2.358 ± 0.283 **	3.597 ± 0.694 **	2553.495 ± 185.827 **	10.383 ± 2.988
OM + IMP	0.838 ± 0.194 ^##^	1.255 ± 0.141 ^##^	1174.551 ± 102.522 ^##^	9.033 ± 2.477
OM + P2X4 shRNA	0.887 ± 0.188 ^##^	1.432 ± 0.174 ^##^	876.716 ± 164.369 ^##^	9.333 ± 3.921
OM + NC shRNA	2.357 ± 0.495	3.116 ± 0.781	2471.759 ± 141.211	10.583 ± 3.187

After the treatment of P2X4 shRNA or imperatorin, the increased levels of serum lipids in obese rats were significantly inhibited. *n* = 10 in each group; values are mean ± SD. The experiments were repeated six times independently ** *p* < 0.01 vs. Control; ^##^ *p* < 0.01 vs. OM.

## Data Availability

The datasets generated and/or analyzed during the current study are available from the corresponding author on reasonable request.
